# *FAM225B* Is a Prognostic lncRNA for Patients with Recurrent Glioblastoma

**DOI:** 10.1155/2020/8888085

**Published:** 2020-11-22

**Authors:** Junsheng Li, Qian Zhang, Peicong Ge, Chaofan Zeng, Fa Lin, Wen Wang, Jizong Zhao

**Affiliations:** ^1^Department of Neurosurgery, Beijing Tiantan Hospital, Capital Medical University, China; ^2^China National Clinical Research Center for Neurological Diseases, China; ^3^Center of Stroke, Beijing Institute for Brain Disorders, China; ^4^Beijing Key Laboratory of Translational Medicine for Cerebrovascular Disease, China; ^5^Beijing Translational Engineering Center for 3D Printer in Clinical Neuroscience, China; ^6^Savaid Medical School, University of the Chinese Academy of Sciences, China

## Abstract

**Objective:**

The overall survival of patients with recurrent glioblastoma (rGBM) is quite different, so clinical outcome prediction is necessary to guide personalized clinical treatment for patients with rGBM. The expression level of lncRNA *FAM225B* was analyzed to determine its prognostic value in rGBMs.

**Methods:**

We collected 109 samples of Chinese Glioma Genome Atlas (CGGA) RNA sequencing dataset and divided into training set and validation set. Then, we analyzed the expression of *FAM225B*, clinical characteristics, and overall survival (OS) information. Kaplan-Meier survival analysis was used to estimate the OS distributions. The prognostic value of *FAM225B* in rGBMs was tested by univariate and multivariate Cox regression analyses. Moreover, we analyzed the biological processes and signaling pathways of *FAM225B*.

**Results:**

We found that *FAM225B* was upregulated in rGBMs (*P* = 0.0009). The expression of *FAM225B* increased with the grades of gliomas (*P* < 0.0001). The OS of rGBMs in the low-expression group was significantly longer than that in the high-expression group (*P* = 0.0041). Similar result was found in the training set (*P* = 0.0340) and verified in the validation set (*P* = 0.0292). In multivariate Cox regression analysis, *FAM225B* was identified to be an independent prognostic factor for rGBMs (*P* = 0.003). Biological process and KEGG pathway analyses implied *FAM225B* mainly played a functional role on transcription, regulation of transcription, cell migration, focal adhesion, etc.

**Conclusions:**

*FAM225B* is expected to be as a new prognostic biomarker for the identification of rGBM patients with poor outcome. And our study provided a potential therapeutic target for rGBMs.

## 1. Introduction

As the most common intracranial malignant tumor in adults [1, 2], glioblastoma (GBM) has a median overall survival (OS) of only 14.6 months [3]. Most GBMs will relapse after standard treatment such as surgical resection, radiotherapy, and chemotherapy [4–6]. In recurrent glioblastoma (rGBM) cases, the patients may lose the opportunity for surgery because of the invasion to the functional area by tumors [7]. And rGBMs are less sensitive to chemotherapy or radiotherapy than primary ones as well [8–10]. So progress in prognostic biomarker identification is required for personalized treatment of patients with rGBM [11]. Further understanding the molecular factors of rGBMs will provide novel insights for the potential biological characteristics and elucidate the possible therapeutic targets.

Long noncoding RNAs (lncRNAs) refer to noncoding RNAs more than 200 nucleotides in length [12, 13] and are associated with a series of cellular process [14–16], such as epigenetic regulation and transcriptional regulation. The dysregulated lncRNAs can be used as prognostic factors for patients [17–20].

In this study, we collected 109 rGBM samples from the Chinese Glioma Genome Atlas (CGGA, http://www.cgga.org.cn/) RNA sequencing dataset. We identified a new prognostic lncRNA *FAM225B* in rGBMs. We investigated the expression patterns of *FAM225B* and evaluated its prognostic value. The expression of *FAM225B* increased with the glioma grades, and it indicated the poor prognosis in rGBM patients. Therefore, *FAM225B* could be a prognostic indicator and a potential therapeutic target for rGBMs.

## 2. Materials and Methods

### 2.1. Patients and Datasets

Our study obtained 109 rGBM samples from the CGGA RNA sequencing dataset with expression data and clinical information [21, 22]. All samples were initially diagnosed with primary glioblastoma (pGBM) and relapsed after standard treatment. The cases were randomly divided into training set and validation set without bias [23]. And the median relative expression of *FAM225B* was used as the cut-off value to divide the samples into a low-expression group and high-expression group. All these samples were diagnosed histologically by 2 neuropathologists according to the 2016 WHO classification guideline of nervous system tumors. Our study was approved by the Ethics Committee of Beijing Tiantan Hospital.

### 2.2. Statistical and Bioinformatic Analysis

SPSS software (version 22; SPSS Inc., Chicago, IL, USA) and R programming language (version 3.2.3) were used for statistical analyses. Survival distributions were estimated by using Kaplan-Meier survival analysis (GraphPad Software Inc., La Jolla, CA, USA). Univariate and multivariate Cox regression analyses evaluated the hazard ratios (HRs) of different prognostic factors and were used to identify the independent prognostic factor. A two-sided *P* value less than 0.05 was set to be statistically significant. Biological process and Kyoto Encyclopedia of Genes and Genomes (KEGG) analysis were conducted by using DAVID (the Database for Annotation, Visualization and Integrated Discovery, https://david-d.ncifcrf.gov/home.jsp) [24, 25].

## 3. Results

### 3.1. *FAM225B* Was Dysregulated in rGBMs

**Figure 1:**
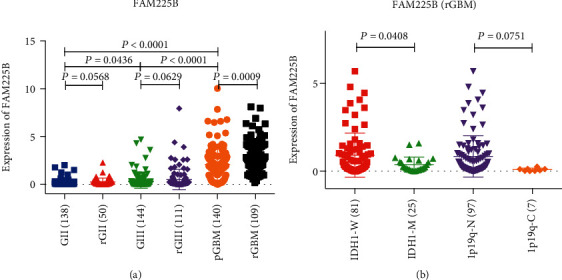
The expression pattern of *FAM225B* in gliomas: (a) *FAM225B* expression in different grades (grade II to grade IV) and types (primary or recurrent); (b) *FAM225B* expression in different IDH1 status and 1p/19q status.

We first investigated the expression pattern of *FAM225B*. The expression of *FAM225B* showed significant difference between the pGBMs and rGBMs (*P* = 0.0009, Figure 1(a)). Moreover, the expression of *FAM225B* increased with grades of gliomas (*P* = 0.0436; *P* < 0.0001; *P* < 0.0001, Figure 1(a)). Then, we compared the *FAM225B* expression level in primary and recurrent gliomas of grade II (*P* = 0.0568) and grade III (*P* = 0.0629, Figure 1(a)).

The IDH1 and 1p/19q status is related to the prognosis of gliomas [26–29]. Therefore, we explored the expression pattern of *FAM225B* in the subtypes of rGBMs. The result showed that the expression of *FAM225B* enhanced in the IDH1-wild-type subtype (*P* = 0.0408, Figure 1(b)). However, no statistically significant differences were observed in the expression of *FAM225B* in rGBM patients with 1p/19q-codeletion or 1p/19q-noncodeletion (*P* = 0.0751, Figure 1(b)).

### 3.2. *FAM225B* Predicted Poorer Overall Survival in rGBMs

All 109 rGBM samples were randomly divided into training set and validation set with no significant data bias. By using the median relative expression of *FAM225B* as the cut-off value, samples were divided into two categories including the low-expression group and high-expression group. Kaplan-Meier analysis was used to investigate the correlation between *FAM225B* expression and OS of rGBM patients.

**Figure 2:**
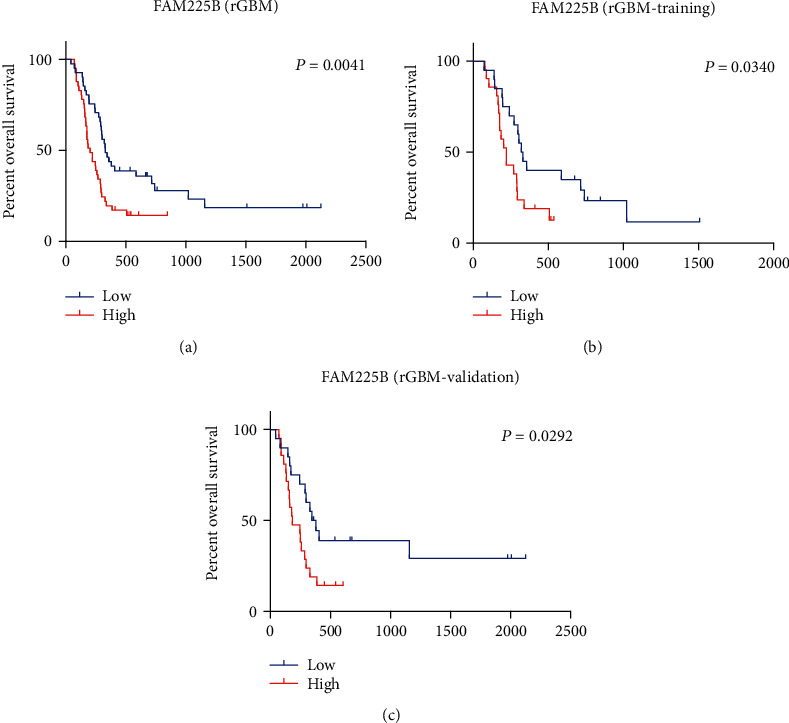
Kaplan-Meier curves of OS among rGBM patients from different groups stratified by the expression of *FAM225B*: (a) Kaplan-Meier curves of OS among rGBMs with different *FAM225B*-expressing levels (high-expression group and low-expression group); (b) Kaplan-Meier curves of OS among rGBMs with different *FAM225B*-expressing levels in the training set (high-expression group and low-expression group); (c). Kaplan-Meier curves of OS among rGBMs with different *FAM225B*-expressing levels in the validation set (high-expression group and low-expression group).

We found that the OS of patients in the low-expression group were significantly longer (*P* = 0.0041, Figure 2(a)). In the training group, the OS of patients in the high-expression group were shorter than those in the low-expression group (*P* = 0.0340, Figure 2(b)). And similar result was observed in the validation set (*P* = 0.0292, Figure 2(c)).

### 3.3. Associations between *FAM225B* and Clinicopathologic Features

**Figure 3:**
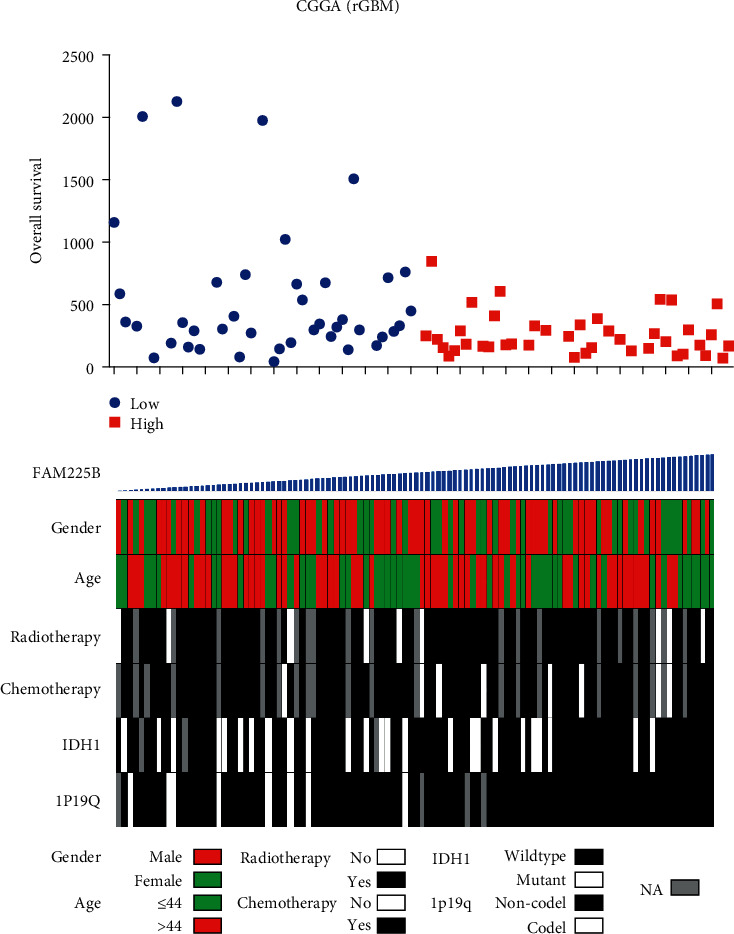
Distribution of clinicopathologic features according to the expression of *FAM225B*. Rows represent corresponding genes, while columns indicate corresponding patients.

We analyzed the relationship between *FAM225B* expression and clinicopathologic features (Figure 3). The results were obtained by the univariate and multivariate Cox regression analyses (Table 1). The univariate Cox regression analysis found that chemotherapy (*P* = 0.003) and *FAM225B* (*P* = 0.007) were potential survival predictors. On the multivariate Cox regression analysis, it showed that *FAM225B* (*P* = 0.003) was an independent prognostic factor. *FAM225B* was significantly related to the prognosis of rGBMs.

### 3.4. Functional Annotation of *FAM225B*

**Figure 4:**
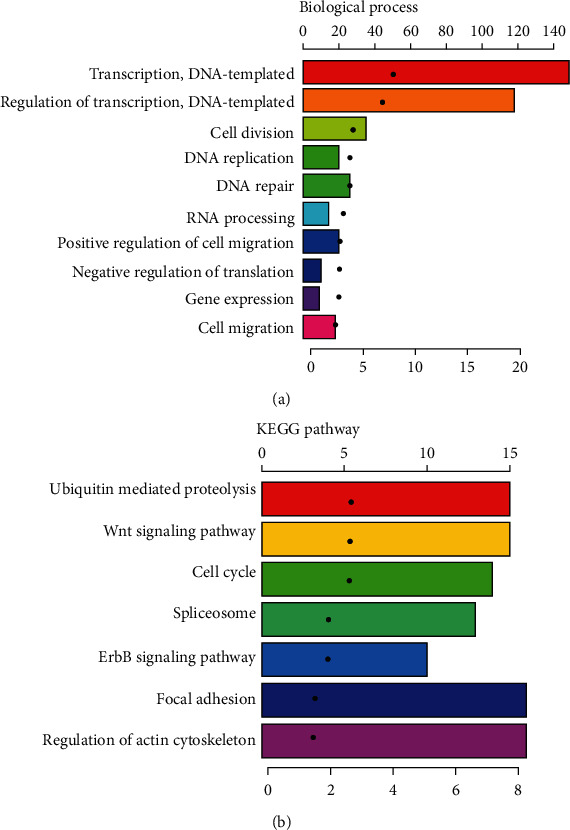
Functional annotation of *FAM225B* in rGBM patients: (a) biological process analysis of the related genes; (b) KEGG pathway analysis of the related genes.

To explain the different prognoses of rGBMs divided by *FAM225B*, we extracted 902 related genes using Pearson correlation analysis (correlation value 0.4; *P* < 0.001). The biological process and KEGG pathway analyses of *FAM225B* were performed using DAVID. It showed that major biological processes were enriched in transcription, regulation of transcription, cell division, positive regulation of migration, cell migration, etc. (Figure 4(a)). KEGG pathway analysis showed that selected genes were enriched in ubiquitin-mediated proteolysis, Wnt signaling pathway, cell cycle, focal adhesion, regulation of actin cytoskeleton, etc. (Figure 4(b)).

## 4. Discussion

Despite great efforts on the multimodal diagnosis and treatment of rGBMs, the clinical prognosis for patients remains poor due to the proliferation potential and antiapoptosis characteristics of this malignant tumor [30–32]. Therefore, understanding the expression of key factors may significantly influence the development and clinical outcome of rGBM patients. Recent studies have found that the dysregulated lncRNAs can provide insights for glioma diagnosis, prognosis, and treatment strategy [33–37].

In this study, we established a dataset of 109 rGBMs collected from the CGGA RNA sequencing dataset to explore the effect of lncRNAs on the prognosis of rGBM patients. By comparing the expression level of *FAM225B* between pGBMs and rGBMs, we found that *FAM225B* expression enhanced in rGBMs (*P* = 0.0009). It indicated that *FAM225B* may play a key role in the recurrence process of GBMs. Then, we analyzed the expression of *FAM225B* in different grades of glioma, including grade II, grade III, and GBM (*P* < 0.0001). The result showed that the expression of *FAM225B* was positively correlated with the grade of gliomas. This indicated that *FAM225B* was associated with glioma malignancy and may serve as a potential indicator for glioma grade. In addition, we explored that the expression of *FAM225B* enhanced in IDH1-wild type (*P* = 0.0408). Based on the data analysis above, we inferred that *FAM225B* can be used as a specific diagnostic marker for GBM recurrence.

We explored the correlation between *FAM225B* expression and survival distribution of rGBM patients. It showed that the OS decreased with the enhancement of *FAM225B* expression (*P* = 0.0041). *FAM225B* enhancement may predict the poor prognosis in rGBMs. We analyzed the relationship between *FAM225B* expression and clinicopathologic features. It indicated that *FAM225B* expression could be a significantly independent prognostic maker in rGBMs.

In our studies, functional annotation analysis showed a strong correlation between *FAM225B* and transcription. It suggested that *FAM225B* may play an important role in rGBM through transcription and its regulation. By using the starBase website (https://starbase.sysu.edu.cn/), we found the candidate miRNAs (miR-1-3p, miR-206, and miR-205-5p) which may be correlated to *FAM225B*. *FAM225B* could competitively bind with miR-1-3p and miR-206, inducing the upregulation of AXL. It may lead to the enhancement of drug resistance and cell proliferation in glioma [38]. Furthermore, it has reported that miR-205-5p is involved in Wnt signaling, cell cycle, and focal adhesion [39], which is consistent with our KEGG findings. These processes may be associated with the recurrence of GBM. However, there are a few limitations in our study. The sample size of our dataset is still limited. Moreover, this is a retrospective study, and different treatments may have an impact on patient survival outcomes. And these findings were based on bioinformatic analysis. Prospective study is needed to clarify the mechanism of *FAM225B* and to further validate the clinical application in patients with rGBMs.

## 5. Conclusion

Our study found that *FAM225B* enhanced in rGBMs and its upregulation was related to the poor prognosis of rGBM patients. Therefore, our study provides a new perspective for *FAM225B*, as a prognosis biomarker and a potential therapeutic target for rGBMs. Further study will investigate the mechanisms of *FAM225B* in rGBMs.

## Figures and Tables

**Table 1:** Univariate and multivariate Cox regression analysis of survival in rGBMs.

Items	Univariate Cox		Multivariate Cox	
	*p* value	HR	*p* value	HR
Gender	0.415	0.811		
Age	0.286	1.010		
IDH1	0.083	0.645		
1p/19q	0.100	0.375		
Radiotherapy	0.102	1.805		
Chemotherapy	0.003	0.256	0.003	0.257
*FAM225B*	0.007	1.279	0.003	1.313

Gender: male, female; IDH1 status: wild type, mutant; 1p/19q status: noncodeletion, codeletion; radiotherapy: no, yes; chemotherapy: no, yes.

## Data Availability

All CGGA data used in this study was available from the CGGA website (http://www.cgga.org.cn).
